# Home consumption of two fortified balanced energy protein supplements by pregnant women in Burkina Faso

**DOI:** 10.1111/mcn.13134

**Published:** 2021-01-06

**Authors:** Brenda de Kok, Katie Moore, Leslie Jones, Katrien Vanslambrouck, Laeticia Celine Toe, Moctar Ouédraogo, Rasmané Ganaba, Saskia de Pee, Juliet Bedford, Carl Lachat, Patrick Kolsteren, Sheila Isanaka

**Affiliations:** ^1^ Department of Food Technology, Safety and Health, Faculty of Bioscience Engineering Ghent University Ghent Belgium; ^2^ Anthrologica Oxford UK; ^3^ Institut de Recherche en Sciences de la Santé (IRSS), Unité Nutrition et Maladies Métaboliques Bobo‐Dioulasso Burkina Faso; ^4^ AFRICSanté Bobo‐Dioulasso Burkina Faso; ^5^ Applying Evidence for Nutrition (AE4N) Wassenaar The Netherlands; ^6^ Nutrition Division World Food Programme Rome Italy; ^7^ Division of Food and Nutrition Policy and Programs Friedman School of Nutrition Science and Policy, Tufts University Boston Massachusetts USA; ^8^ Departments of Nutrition and Global Health and Population Harvard T.H. Chan School of Public Health Boston Massachusetts USA

**Keywords:** acceptability, balanced energy protein (BEP) supplements, Burkina Faso, cultural context, low birth weight, low‐income countries, maternal nutrition, nutritional interventions, pregnancy outcome

## Abstract

Balanced energy protein (BEP) supplementation for pregnant and lactating women in low‐ and middle‐income countries is a promising strategy to improve birth outcomes and child growth. The objective of this study was to assess and compare the acceptability of new formulations of two fortified BEP supplements, a lipid‐based peanut paste and a vanilla biscuit, among 80 pregnant women in rural Burkina Faso, prior to an efficacy trial. A 10‐week individually randomized cross‐over study was designed, in which women received a weekly supply of each supplement for 4 weeks, and a daily choice between the supplements in the last 2 weeks. Questionnaires to assess daily consumption and supplement acceptability (*n* = 80) and home observations (*n* = 20) were combined with focus group discussions (*n* = 6) and in‐depth interviews with women (*n* = 80) and stakeholders (*n* = 24). Results showed that the two supplements were well accepted. Quantitative findings indicated high compliance (>99.6%) and high overall appreciation (Likert score >6 out of 7) of both supplements. The assessment of preferred choice in Weeks 9 and 10 indicated a slight preference for the vanilla biscuit. Qualitative findings indicated that perceived health benefits, support from household members and educational messages from health professionals were important drivers for acceptance and compliance. Sharing was not often reported but was identified during interviews as a possible risk. We recommend that future studies use a combination of methods to identify appropriate food supplements and context‐specific factors that influence acceptability, compliance and subsequent impact of nutritious food supplements.

**Key messages**

- The findings illustrate that both the lipid‐based peanut paste and the vanilla biscuit were well accepted by pregnant women in Burkina Faso during a 10‐week home feeding trial.
- Information on the use and benefits of nutritious food supplements by health care professionals, provided in a systematic and understandable manner, and engaging community leaders and family members appeared to be important factors for acceptance and compliance.
- A mixed‐method acceptability study is a valuable investment prior to an efficacy trial to examine context‐specific drivers and barriers to supplement use, develop strategies to increase compliance and improve quality of study findings.

## 1 INTRODUCTION

Low birth weight (LBW) and small‐for‐gestational age (SGA) affect an estimated 20 to 30 million infants every year (Katz et al., 2013; Lee et al., 2013). These adverse birth outcomes lead to an increased risk of neonatal and infant mortality and can also have long‐term consequences by increasing the risk of developing metabolic syndrome (Lawn, Cousens, Zupan, & Team, 2005; Lee et al., 2017; Levy‐Marchal & Jaquet, 2004; Saenger, Czernichow, Hughes, & Reiter, 2007). It is well known that poor maternal nutritional status can contribute to this restricted foetal growth (approximated by LBW and SGA) and is a major problem in many low‐income countries (Cetin, Mando, & Calabrese, 2013; da Silva Lopes et al., 2017; Gernand, Schulze, Stewart, West, & Christian, 2016).

To improve nutritional status, the World Health Organization (WHO) recommends balanced energy protein (BEP) dietary supplementation during pregnancy in undernourished populations to reduce the risk of stillbirths and SGA newborns (WHO, 2016). Although recent studies have demonstrated a positive effect of BEP supplements in pregnant women on birth outcomes, authors highlight the limited amount of available evidence and the need to evaluate the effect of this balanced supplement by large, well‐designed randomized trials (Imdad & Bhutta, 2012; Ota, Hori, Mori, Tobe‐Gai, & Farrar, 2015; Stevens et al., 2015). Such studies will help to generate more robust evidence on the impact to guide recommendations and decision making. For this reason, the MISAME‐III (MIcronutriments pour la SAnté de la Mère et de l'Enfant) study was designed. The study is a randomized controlled trial to assess the effect of a fortified BEP supplement for pregnant and lactating women on birth and child health outcomes (ClinicalTrials Identifier: NCT03533712).

The results of the efficacy trial will depend to a large extent on the optimal use of the BEP supplements. In comparison with fortified blended foods (FBFs) for pregnant and lactating women, BEP supplements provide the advantage that there is no preparation required. Although FBFs are often regarded as family foods, little is known about what type of ready‐to‐use product(s) pregnant and lactating women appreciate and how they are used. Understanding the factors that determine the acceptability of BEP supplements is however crucial to reduce the risk of poor compliance (Iuel‐Brockdorf et al., 2016; Janmohamed et al., 2015; Young, Blanco, Hernandez‐Cordero, Pelto, & Neufeld, 2010). Hence, prior to testing the efficacy in the main trial, a formative study to assess acceptability and utilization of BEP supplements was designed. Twelve supplements of different types and flavours were rapidly assessed in terms of short‐term acceptability using a single‐meal test to inform the present study (Jones et al., 2020). Here, we evaluate the two highest‐ranking supplements from the meal test to assess (1) medium‐term acceptability (defined for the present study as 4–6 weeks), (2) compliance during at‐home consumption and (3) influencing factors for acceptability and compliance.

## 2 MATERIALS AND METHODS

### 2.1 BEP supplements

An expert consultation was held at the Bill and Melinda Gates Foundation (BMGF, 2017) to provide guidance on the optimal nutritional composition and formulation of new BEP supplements for use in pregnant and lactating women. The guiding principles emphasize that a BEP supplement is fortified and thus provides a combination of multiple micronutrients, energy and protein in a balanced combination. This is in agreement with a recent conclusion that multiple micronutrient supplements can reduce the risk of adverse birth outcomes compared with iron and folic acid alone (Bourassa et al., 2019). For this study, two forms of fortified BEP supplements were manufactured: a lipid‐based peanut paste with a single serving of 72 g (389 kcal; 14.5‐g protein) and a vanilla biscuit, served in a package of six biscuits, with a total portion size of 75 g (375 kcal; 16.5‐g protein). The nutritional content of both supplements is compatible with the macronutrient and micronutrient targets developed during the BMGF expert consultation. Levels of folic acid and iron in the BEP supplements fall within the recommended intake range by WHO (2016) and do not reach, even when combined with iron–folic acid tablets, the upper intake levels for folic acid.

### 2.2 Study design

**FIGURE 1:**
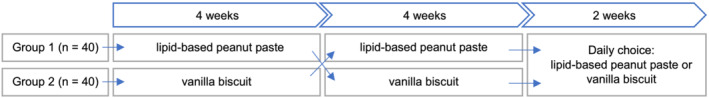
Study design

The study followed a 2‐arm cross‐over design, testing the two BEP supplements for a period of 10 weeks (Figure 1). During the initial 8‐week period, each of the two supplements were utilized for 4 weeks, and in the subsequent 2 weeks, a daily choice between the lipid‐based peanut paste and vanilla biscuit was offered to all women. Women were individually randomized to determine which of the two supplements they would use first, the lipid‐based peanut paste (Group 1) or the vanilla biscuit (Group 2). A random allocation scheme was developed in Excel, and for each of the two health centres, 20 women were assigned to either Group 1 or 2 by drawing an envelope containing the random group assignment.

### 2.3 Study area and participants

The study was conducted from December 2018 to March 2019 in Houndé, the district capital of the province of Tuy in the midwest area of Burkina Faso. In this landlocked country in West Africa, a substantial portion of women are underweight and the neonatal and under‐five mortality rate was high compared with global rates in 2018 (INSD & ICF International, 2012; UN IGME, 2018). Previous research in Tuy Province reported high levels of LBW (around 14%) and SGA (between 32% and 42%), indicating a need for action in this area (Huybregts et al., 2009; Roberfroid et al., 2008).

Houndé has one district hospital and 31 health centres (HC). Two health centres (Boni and Kari) were selected for this formative study on the basis of accessibility and are representative of the broader study area in terms of ethnicity and religion. A convenience sample of 80 pregnant women attending antenatal services at the selected health centres were invited to participate in collaboration with the health centre officer‐in‐charge. Women had to be pregnant and aged between 15 and 40 for inclusion, which is in line with the inclusion criteria for the efficacy trial. Women were excluded if they reported an allergy to soy, dairy products, eggs, gluten or peanuts.

### 2.4 Data collection

A set of quantitative and qualitative research tools was developed to assess medium‐term acceptability and consumption in the context of Burkina Faso. The French tools were translated into Mooré and Dioula with input from data collectors during a 2‐week training and were refined based on feedback from the field research partner AFRICSanté. Prior to data collection, tools were pilot tested with pregnant women and revised for clarity prior to implementation.

#### 2.4.1 Quantitative tools

##### Demographic questionnaire (*n* = 80)

An interview‐based questionnaire was used to collect basic sociodemographic (age, school level, etc.) and obstetric history information.

##### Supplement acceptability questionnaire (*n* = 80)

Hedonic properties were assessed using a structured questionnaire at the end of Week 4 and Week 8. Women were asked to rate the supplement's colour, taste, texture, smell and overall appreciation using a 7‐point Likert scale, ranging from 1 (*dislike very much*) to 7 (*like very much*). Subsequently, women were asked to respond to 11 statements regarding their perception of current and future supplement use during pregnancy. Responses were scaled from 1 (*I don't agree at all*) to 7 (*I agree completely*) when asked for their opinion. The scale was complemented with a range of emoticon faces (very unhappy to very happy), which has been used elsewhere to measure food acceptability in illiterate populations (Cohuet et al., 2012; Hess et al., 2011; Isanaka et al., 2019; Iuel‐Brockdorf et al., 2015).

##### Daily consumption questionnaire (*n* = 80)

Women's consumption behaviour, including supplement choice in Weeks 9 and 10, was quantitatively assessed using a short multiple‐choice questionnaire on a daily basis at home. Nine questionnaire items assessed the portion size consumed, timing, meal replacement and sharing. If the supplement was not consumed, the reason was registered.

##### Home observations (*n* = 20)

At‐home consumption was directly observed twice among a random subsample of 20 women (10 per study arm) during Weeks 3 and 4 and Weeks 6 and 7. Participant activities were registered on paper using a standard set of codes every 5 min during 12‐consecutive hours. All food‐related activities (eating or drinking, preparing or cooking food, buying or storing food) and observations concerning the supplement during the 12‐h observation period were described in more detail in an open text comment box. In case the participant left the house during this period, the home observer asked permission to follow her during the activities outside the household.

#### 2.4.2 Qualitative tools

##### In‐depth interviews (*n* = 80)

To identify factors influencing acceptability and consumption of the supplements, semistructured interviews were held with women at four times over the course of the at‐home tasting period: Week 1 (initial interview), Week 4 (supplement‐specific interview 1), Week 8 (supplement‐specific interview 2) and Week 10 (final interview with discussion of supplement choice). The interview guide contained general questions on household food practices and beliefs; diet during pregnancy and lactation, availability and access of supplements, and antenatal care‐seeking behaviour; and supplement‐specific questions exploring attitudes and user experience (Table S1).

##### In‐depth interviews with stakeholders (*n* = 24)

To identify stakeholders' attitudes towards supplementation during pregnancy, interviews were held with family members (*n* = 8), health professionals (*n* = 8) and community leaders (*n* = 8) in Week 6. Semistructured interview guides were adapted to each stakeholder group.

##### Focus group discussions (FGDs) (*n* = 6)

To elicit additional information on women's experience with the BEP supplements and factors that influence acceptability and compliance, six FGDs (each with eight women randomly selected from our study sample) were held using a semistructured thematic guide in Week 10 (Table S2).

### 2.5 Procedures

Before the start of 10‐week home feeding trial, women were visited at home to receive information about the study, as well as the risks, benefits and use of the supplements. The voluntary, confidential and anonymous nature of participation was emphasized. Informed consent was then obtained, and a sociodemographic questionnaire was administered.

During the first 8 weeks, a 1‐week supply of supplements was given to the pregnant women at home. Trained village women visited participants daily to complete the ‘Daily Consumption Questionnaire’ and collect the empty sachets. During the last 2 weeks, the procedure was similar except that participants were given a daily choice to receive either the lipid‐based peanut paste or vanilla biscuit. This daily choice was offered by the trained village women who had a supply of both products with them at each home visit. In a subsample, home observations were conducted from 6 am to 6 pm by teams of two data collectors, each observing for a 6‐h shift.

The interviews were held either at home (for pregnant women, family members and community leaders) or at the health centre (for health professionals) and usually lasted no more than 60 min. The FGDs were held at the health centre, led by one facilitator assisted by a note taker and lasted approximately 2–2.5 h. All interviews and FGDs were audio recorded and transcribed from local languages into French. In general, efforts were made to ensure the place used for data collection activities was as private and neutral as possible. The field team was familiar with the local context and languages use in each field site, and participants were encouraged to speak in the language in which they were most comfortable and confident.

### 2.6 Data analysis

Quantitative data were collected electronically using CSPro (version 7.3.1). The CSPro data files were exported to STATA 14.2 (StataCorp, College Station, TX) and R software for statistical analysis. Data are presented as median and interquartile range (Q1–Q3) or counts and percentages. Likert scales used in the ‘Supplement Acceptability Questionnaire’ were treated as continuous variables (Sullivan & Artino, 2013). Presence of a carryover effect due to the crossover study design was tested by computing carry‐over, period, sequence and treatment effect using the pkcross STATA command. All registered activity codes from the home observation tool were copied to Excel and imported into R to calculate the counts and percentages.

For the interviews and FGDs, a framework with the most apparent themes was developed through systematic review of the transcripts (Sargeant, 2012). In the next step, a codebook was developed in Dedoose analytic software (version 8.2.32; SocioCultural Research Consultants), and all transcripts were imported for analysis by labelling phrases within the transcripts. A selection of transcripts was reviewed by two separate researchers in order to cross‐check codes and ensure consistency.

The results of the quantitative and qualitative methods were compared for a comprehensive analysis on the medium‐term acceptability of the lipid‐based peanut paste and vanilla biscuit. This allowed for triangulation of results on women's compliance, preference, contextual utilization and opinions on future use and messages (Harris et al., 2009).

### 2.7 Ethical considerations

Permission to undertake this study was granted by Ghent University Hospital Ethics Committee, the Comité d'éthique Institutionnel du Centre Muraz in Burkina Faso and the Harvard T.H. Chan School of Public Health Institutional Review Board. Participants provided written, informed consent prior to enrolling in the study.

## 3 RESULTS

A total of 80 pregnant women participated in the 10‐week home feeding trial, of which 28 women gave birth during the study period. All women completed the full study period. Demographic and socio‐economic characteristics are presented in Table 1. In addition, eight health professionals, eight family members (four husbands, three mothers‐in‐law and one brother‐in‐law) and eight community leaders were interviewed.

**TABLE 1:** Characteristics of study participants

Characteristics	Total sample pregnant women (*n* = 80)	Randomization group 1 (*n* = 40)	Randomization group 2 (*n* = 40)
Age (mean ± SD)	26.3 ± 6.0	26.3 ± 5.9	26.3 ± 6.1
Matrimonial status, *n* (%)			
Married	76 (95%)	38 (95%)	38 (95%)
Cohabitation	2 (2.5%)	2 (5%)	0 (0%)
Not married	2 (2.5%)	0 (0%)	2 (5%)
School attendance, *n* (%)			
None	52 (65%)	23 (57.5%)	29 (72.5%)
Primary	17 (21%)	11 (27.5%)	6 (15%)
Secondary	11 (14%)	6 (15%)	5 (12.5%)
Higher education	0 (0%)	0 (0%)	0 (0%)
Household size, number of people (mean ± SD)	6.2 ± 3.4	5.9 ± 3.9	6.5 ± 3.0
Household size, number of children <5 years old	1.2 ± 1.1	1.2 ± 1.0	1.2 ± 1.1
Village, *n* (%)			
Boni	40 (50%)	20 (50%)	20 (50%)
Kari	40 (50%)	20 (50%)	20 (50%)
Religion, *n* (%)			
Animist	34 (43%)	15 (37.5%)	19 (47.5%)
Christian	25 (31%)	14 (35%)	11 (27.5%)
Muslim	21 (26%)	11 (27.5%)	10 (25%)
Gestational age in months (mean ± SD)	5.4 ± 1.9^a^	5.5 ± 1.9	5.4 ± 2.0
First pregnancy, *n* (%)	18 (23%)	10 (25%)	8 (20%)
At least one previous foetal death, *n* (%)	11 (14%)	5 (12.5%)	6 (15%)
At least one previous child death, *n* (%)	15 (19%)	10 (25%)	5 (12.5%)
Number of children (mean ± SD)	1.9 ± 1.7	1.8 ± 1.8	2.0 ± 1.6
Number of children <5 years old	0.6 ± 0.6	0.7 ± 0.6	0.5 ± 0.5
Number of pregnancy consultations (mean ± SD)	1.5 ± 1.1	1.7 ± 1.1	1.3 ± 1.0

*Note*: Eight women did not know their gestational age.

^a^ Mean ± SD for 72 women.

The quantitative and qualitative research methods produced largely consistent and convergent results that both the lipid‐based peanut paste and vanilla biscuit were well accepted. Results on the main study objectives (1) evaluation and preference, (2) compliance and (3) influencing factors of the BEP supplements are presented below.

### 3.1 Evaluation and preference

Both BEP supplements were positively evaluated. There was no difference in appreciation, convenience or intended daily use between the lipid‐based peanut paste and vanilla biscuit after a consumption period of 4 weeks (Table 2). Both supplements scored ≥6 on a 7‐point Likert scale. A positive association between the supplements and other familiar foods such as peanuts, milk and chocolate appeared to influence both use and preference. Furthermore, participants gave a median score of 6 (*I agree*) out of 7 on the statement whether the supplements are a medicine (Table 2).

**TABLE 2:** Acceptability of BEP supplements at Week 4 and Week 8

	Lipid‐based peanut paste			Vanilla biscuit		
	All (*n* = 80)	Group 1 (*n* = 40)	Group 2 (*n* = 40)	All (*n* = 80)	Group 1 (*n* = 40)	Group 2 (*n* = 40)
Appreciation of supplement (1 = *I dislike it very much* to 7 = *I like it very much*), median (Q1–Q3)						
Colour	7 (6–7)	7 (7–7)	7 (6–7)	7 (6–7)	7 (6–7)	7 (7–7)
Taste	6 (6–7)	6 (6–7)	6 (6–7)	7 (6–7)	7 (6–7)	6.75 (7–7)
Texture/consistency	6 (6–7)	6 (5–7)	6.5 (6–7)	7 (6–7)	7 (6–7)	7 (6–7)
Smell	6 (3–7)	6 (3.75–7)	5.5 (2–7)	7 (6–7)	7 (6–7)	7 (7–7)
Overall appreciation	6 (5–7)	6 (5–7)	6 (5–7)	7 (6–7)	7 (6–7)	7 (6.75–7)
Perceived child likeability	7 (6–7)	7 (6–7)	7 (6–7)	7 (6–7)	7 (7–7)	6.5 (6–7)
Perceived adult likeability	6 (5–7)	6 (5–6.75)	6 (5–7)	6 (4–7)	6 (6–7)	6 (4–7)
Perception of supplement use (1 = *I don't agree at all* to 7 = *I agree completely*), median (Q1–Q3)						
Supplement is convenient to eat	6 (6–7)	6 (6–7)	6.5 (6–7)	7 (6.75–7)	7 (6–7)	7 (7–7)
Supplement is convenient to eat between meals	6 (5–7)	6 (5.75–7)	6 (4.75–7)	6 (5–7)	6 (6–7)	6 (5–7)
Supplement is medicine	6 (3.75–6)	5 (3–6)	6 (5–7)	6 (5–7)	6 (4.5–7)	7 (5–7)
Feel full after full portion	5 (3–6)	5 (3–6)	5 (3–6)	5 (3–6.25)	5 (3–6)	6 (5–7)
Would share with others	1 (1–2.25)	1 (1–2)	2 (1–3)	1 (1–3)	1 (1–2)	1 (1–3.25)
Willingness to use daily for 12 months (1 = *I don't agree at all* to 7 = *I agree completely*), median (Q1–Q3)						
Would use if provided	7 (6–7)	6.5 (6–7)	7 (6–7)	7 (6–7)	7 (6–7)	7 (6.75–7)
Would use if purchased	5 (3–7)	5 (3.75–7)	6 (3–7)	6 (4–7)	5.5 (5–7)	6 (3–7)
Would pay how much, per daily dose (CFA)^a^, n (%)						
0–100	47 (58.7)	24 (60)	23 (57.5)	58 (72.5)	30 (75)	28 (70)
101–200	16 (20)	8 (20)	8 (20)	13 (16.3)	7 (17.5)	6 (15)
201–300	3 (3.8)	1 (2.5)	2 (5)	5 (6.2)	1 (2.5)	4 (10)
301–400	0 (0)	0 (0)	0 (0)	0 (0)	0 (0)	0 (0)
401–500	6 (7.5)	5 (12.5)	1 (2.5)	2 (2.5)	2 (5)	0 (0)
>500	1 (1.3)	0 (0)	1 (2.5)	0 (0)	0 (0)	0 (0)
Do not know	7 (8.7)	2 (5)	5 (12.5)	2 (2.5	(0)	2 (5)
Acceptability of portion size (for a snack), *n* (%)						
Portion size is acceptable	75 (93.8)	37 (92.5)	38 (95)	72 (90)	37 (92.5)	35 (87.5)
Too small	1 (1.2)	1 (2.5)	0	3 (3.8)	2 (5)	1 (2.5)
Too big	4 (5)	2 (5)	2 (5)	5 (6.2)	1 (2.5)	4 (10)
Preference for continuation of consumption during pregnancy and during the breast‐feeding period at Week 8, *n* (%)						
Choice of supplement	32 (40)	11 (27.5)	21 (52.5)	48 (60)	29 (72.5)	19 (47.5)

^a^ The exchange rate for CFA during the study period was between 0.0017 and 0.0018 to 1 USD, retrieved from: www.OANDA.com.

The final 2 weeks, where participants were offered a choice in supplements, revealed a slight preference for the vanilla biscuit over the lipid‐based peanut paste. After having tried each product for 4 weeks, 60% of women indicated in the ‘Supplement Acceptability Questionnaire’ that they would opt for the vanilla biscuit during pregnancy and lactation if they were to continue after the 8‐week test period. When the women were offered a daily choice between the two products in Weeks 9 and 10, they also showed a preference for the vanilla biscuit, as it was chosen more often: 63% of all times (705/1120) versus 37% (415/1120) for the lipid‐based peanut paste. The interviews and FGDs confirmed this small preference for the vanilla biscuit. Positive attributes of the vanilla biscuit were mainly the sweet taste and overall appreciation (*I like it*), whereas reasons for choosing the lipid‐based peanut paste included a pleasant feeling in the mouth and easy‐to‐consume. A few women reported an aversion to the smell of the peanut paste as the reason to choose the vanilla biscuit.

### 3.2 Compliance

High compliance to both the lipid‐based peanut paste (99.8% of all servings consumed) and vanilla biscuit (99.6% of all servings consumed) was recorded during the first 8 weeks, as well as during the choice period in Weeks 9 and 10 (lipid‐based peanut paste 100%; vanilla biscuit 99.9%). Only three women missed days of consumption. Of these, one woman missed a period of 12 days because she gave birth. Two women missed a single day, one because she gave birth and one without indicating a reason. The full portion of both supplements was nearly always consumed. During the first 8 weeks, 95.8% of all portions of the lipid‐based peanut paste were consumed in their entirety. In the last 2 weeks (when women specifically chose the lipid‐based peanut paste), this was 99.8%. For the vanilla biscuit, this was 99.6% during the first 8 weeks and 100% during the last 2 weeks. Generally, the supplement was consumed in a single sitting (lipid‐based peanut paste 79%; vanilla biscuit 77%) and in 2/3 of all cases it was consumed as a morning snack. No sequence nor period effect was observed for the cross‐over model, indicating that there was no difference in portion consumed by temporal order of supplement use.

The home observations corroborated the findings on compliance for both supplements. Notes in the open text comment boxes of the home observation tool showed that women consumed the full portion of the supplement in the morning. Results from the interviews and FGDs illustrated factors that could explain this high compliance. Participants commented favourably on portion size, taste and perceived health benefits:

Interviewer “Why did you eat the full portion every day?”

In‐depth interview 2, HC Boni, participant 20 [lipid‐based peanut paste] “I find it the right amount and that is the reason I can finish the package every day.”

FGD 1, HC Boni, participant 8 [vanilla biscuit] “I still love it now like in the beginning because it's good.”

FGD 4, HC Kari, participant 10 [lipid‐based peanut paste] “I consume it because it has been said that it is food for pregnant women, so I know that if I consume it, it will improve my body. That is the reason I consume it every day.”

#### 3.2.1 Compliance intention

Quantitative results showed high willingness to continue consumption of the food supplements during pregnancy and lactation with a median score of 7 (*I agree completely*) (Table 2). Similar results were found in the interviews and FGDs. Only five women specifically said they would find it difficult to continue supplementation throughout their pregnancy and 6 months after delivery because they did not like the supplement.

### 3.3 Factors influencing acceptability and compliance

All study tools, in particular the interviews and FGDs provided information on facilitating factors and barriers for acceptability and compliance: (1) dietary practices and beliefs for pregnant women, (2) perceived health benefits, (3) support from stakeholders, (4) sharing of the supplement, (5) educational messages from health professionals and (6) supplement choice. The combined data is presented per theme below.

#### 3.3.1 Dietary practices and beliefs for pregnant women

Women and other stakeholders uniformly recognized the importance of a healthy diet to ensure the well‐being of a pregnant woman and her foetus. Participants agreed that the quantity of food consumed during pregnancy and lactation should be increased. However, no other changes in the diet, such as intentional reduction of dietary intake to reduce the perceived risk of delivery complications due to large babies, were reported that could negatively influence the acceptance of BEP supplements. This was supported by experiences during the 10‐week at‐home consumption. Pregnant women and other stakeholders reported that the supplement was generally taken in addition to the normal diet and did not replace all or part of a meal. However, a small number of reports indicated that women were at times too full after having eaten the supplement to eat either their normal diet or additional snacks. This was more consistent in the context of the lipid‐based peanut paste rather than the vanilla biscuit.

#### 3.3.2 Perceived health benefits

Feeling good, stronger, healthier; having an improved appearance; and increased appetite were benefits of consuming the supplements identified by pregnant women in this formative study:

FGD 3, HC Boni, participant 7 “When you eat it every day it makes you shine and your child also shines, he is strong and healthy.”

FGD 4, HC Kari, participant 14 “It improved my body posture, it boosted my appetite.”

The perceived benefits for mother and child also appeared the main reason for their intention to continue supplementation:

In‐depth interview 2, HC Kari, participant 11 “I would continue to consume it to be healthy and for my child to be healthy.”

FGD 4, HC Kari, participant 14 “Because by consuming it, it will stimulate the breast milk and if the child sucks, it will have vitamins.”

#### 3.3.3 Support from stakeholders

Family members, community leaders and health professionals expressed their willingness to support the use of the supplements for pregnant women and to encourage heads of households to permit use. The benefits of the supplement for mother and child and the recommendation by health professionals were the most important reasons to provide support. The pregnant women suggested that community leaders and family members, in particular the head of household and/or husband, should be engaged when introducing food supplements as they play an important role in the understanding and acceptance of the supplement.

#### 3.3.4 Sharing of the supplement

Data captured by the quantitative questionnaire indicated that women's intention to share was very low for both supplements (mean Likert score of 1 (1–3) out of 7, Table 2). This finding was confirmed by the home observations in which no sharing was registered (Table 3). Furthermore, during the interviews, the majority of women revealed that they did not share the supplement with other adults or their children. These findings were corroborated by interviews with family members and did not differ between the supplement types. The main factors cited for not sharing included: the fact that the supplement is only intended for pregnant women; it is a medicine; instructions were given not to share; and women's belief that they would otherwise not receive the full benefit of the supplement. Despite the general tendency not to share, a handful of women indicated that they did share a small portion with their child(ren) because they were in their presence while eating. To avoid sharing, some women chose a separate room or took a moment alone to eat the supplement:

In‐depth interview 4, HC Kari, participant 33: “The reason I eat in my room is because of adults and children. If you eat outside, a child will see and cry for you to give it. If you refuse to give, they will say that it is you who is bad. They say not to give to children but there are some who give it to their child.”

**TABLE 3:** Food related activities during 12 h home observation (total number of observations)

	Lipid‐based peanut paste			Vanilla biscuit		
	All (*n* = 40)	Group 1 (*n* = 20)	Group 2 (*n* = 20)	All (*n* = 40)	Group 1 (*n* = 20)	Group 2 (*n* = 20)
Food related activities, *n* (%)						
Eating	299 (5.2)	157 (5.5)	142 (4.9)	319 (5.5)	174 (6.0)	145 (5.0)
Drinking	41 (0.7)	20 (0.7)	21 (0.7)	31 (0.5)	20 (0.7)	11 (0.4)
Preparing or cooking food	507 (8.8)	276 (9.6)	231 (8.0)	511 (8.9)	254 (8.8)	257 (8.9)
Purchasing food	2 (0.0)	2 (0.1)	0 (0.1)	3 (0.1)	2 (0.1)	1 (0.0)
Storing food	10 (0.2)	7 (0.2)	3 (0.0)	12 (0.2)	3 (0.1)	9 (0.3)

*Note*: Each observation represents the registration of an activity at a 5‐min interval; some activities may thus have been recorded as more than one observation.

Women indicated that the instruction not to share the supplement helped to manage household members' expectations. Most participants agreed that family members would accept that sharing is not advised, as long they understand the underlying aim of daily supplementation:

In‐depth interview, community leader 4: “For those who understand things, will understand that it is made for pregnant women. But, for those who do not understand anything, even if the woman says that it is only for her alone, they will say that it is mean not to share with them.”

Despite the majority's willingness to follow the instructions not to share, a small number of women and stakeholders suggested that there is a likelihood of the supplement being shared with children in the future. In general, participants mentioned that both the quantity of the supplement provided and women's personal and subjective approach to utilization and adherence were key determinants of whether or not a snack would be shared with others:

In‐depth interview, community leader 4: “Good! Eh! There are people when they buy, not everyone, and eat that alone. People don't have the same character, that's it! There are others when they buy, they do everything for the family to benefit.”

Strategies to avoid sharing of the BEP supplements in the future focused on consuming the supplement alone and out of sight of others to avoid requests and conflicts. Women mentioned that for those with sufficient means, another option would be to buy alternative snacks for others to avoid sharing the provided supplement. Sensitization of household members and community leaders was put forward as a strategy to avoid sharing:

FGD 3Boni:

Participant 7 “I think it's better to explain to them the purpose of this work so that they understand well, otherwise they can take it without your knowledge, and it will cause other problems.”

Participant 37 “If you explain them well they will understand and not ask you to share.”

#### 3.3.5 Educational messages from health professionals

Women in this study uniformly agreed that the guidance and information provided at the onset was helpful. Especially the positive effects of the supplement ‘good for mother and unborn child’ and ‘it's a medicine and contains vitamins’ were perceived important messages. A small number of women indicated that they would have liked to receive additional information on the ingredients and how to use the supplement. Health professionals gave the suggestion to use pictures in communication to make sure all women understand the message. Both women and other stakeholders indicated that health centres are the most effective channel for providing information about nutritional supplements and for distribution, either through individual one‐to‐one meetings or in group discussions. Stakeholders agreed that further emphasis on promoting the supplements as a medicine would contribute to successful uptake and adherence.

#### 3.3.6 Supplement choice

Data gathered during the daily choice period (Weeks 9 and 10) highlighted that on average, women asked to change supplements two times over the course of the 2‐week choice period. In total, 24 (30%) women did not request a change, 32 (40%) women asked to change one time and 24 (30%) women asked to change two times or more. The qualitative results were inconclusive: during the final in‐depth interviews, a number of women indicated that they would prefer to have a choice between the two supplements for reasons including a desire for variety and the option to choose what they prefer at a given moment, whereas in the FGDs, participants did not express a strong interest in having a choice between supplement types or flavours.

## 4 DISCUSSION

The present study provides evidence for high acceptability among pregnant women in Burkina Faso of two new fortified BEP supplements over a 10‐week period during pregnancy and lactation. The quantitative data showed a positive assessment of hedonic characteristics, ease of use and compliance. The high acceptability and compliance appeared to be primarily influenced by the perceived benefits to the mother and child and household support for supplement use. The majority of women indicated that they did not share the supplement, but qualitative results highlight the possibility that there may be some sharing with children. Results also gave insight that community sensitization and clear instructions by health professionals could play an important role in uptake and compliance.

The results on drivers of supplement use collected during this study are in line with other acceptability studies, in which perceived health benefits for women and their babies appeared to be an important motivating factor for daily consumption (Adu‐Afarwuah, Lartey, Zeilani, & Dewey, 2011; Iuel‐Brockdorf et al., 2016; Janmohamed et al., 2015; Klevor et al., 2016). Moreover, positive attitudes of stakeholders have been found to be an important factor to promote acceptability and sustained use of supplementary foods by pregnant women in a study evaluating the acceptability of corn–soya blend in rural Cambodia (Janmohamed et al., 2015). Although we did not observe large difference in the responses to stratify the results per stakeholder group, the interviews provided valuable information that family members, community leaders and health professionals support the use of BEP supplements. We therefore recommend future studies or programmes to take attitudes of all stakeholders into account when introducing nutritious food supplements.

Similar to another study on lipid‐based nutrient supplements in Burkina Faso, the women perceived both BEP supplements as a medicine. Scholars have previously suggested introducing supplementary foods as a medical treatment to increase adherence and decrease sharing (Iuel‐Brockdorf et al., 2015, 2016). We acknowledge that this could help to promote acceptability and compliance. Overall, the need for clear instructions and educational message is often mentioned as a key element to ensure appropriate utilization (Aguayo et al., 2005; Kodish, Aburto, Hambayi, Kennedy, & Gittelsohn, 2015; Seck & Jackson, 2008). Although the present findings corroborate these previous observations, it remains essential to investigate context‐specific factors that could affect acceptability and compliance.

In this formative study, sharing practices were not apparent and did not differ between the supplement types. In contrast to our findings, various studies report sharing of food supplements (Iuel‐Brockdorf et al., 2016; Janmohamed et al., 2015; Langlois et al., 2020; Lyssenko et al., 2007; Marquer et al., 2020). A reason for this difference could be that sharing is inherently difficult to assess due to the lack of objective measures. Research to better understand social dimensions of food and the optimal dose of supplements remains therefore important to ensure high compliance and reach the desired impact (Demusz, 2000; Pottier, 1996). Hereby, it is important to take into account that in specific cultural contexts and food insecure areas, social and moral pressure to share can be high (Klevor et al., 2016; Kodish, Aburto, Nseluke Hambayi, Dibari, & Gittelsohn, 2016; Lentz, Ouma, & Mude, 2016; Maxwell & Burns, 2008).

The relative large sample‐size, cross‐over design and 10‐week duration are strengths of our study. All women were given each supplement for a period of 4 weeks and offered a choice in the final 2 consecutive weeks, allowing for a thorough evaluation of acceptability. The available evidence suggests that a 4‐week period of daily consumption is sufficient to examine acceptability, as patterns of decreased motivation or sensory satiety appear after 2–3 weeks as a function of the number of times a food is consumed (Clark, Dewey, & Temple, 2010; Liem & Zandstra, 2009; Temple, Chappel, Shalik, Volcy, & Epstein, 2008). However, we extended the study period in order to generate a more rounded comparison between the supplements. Although Likert scale results did not show any substantial difference between the two supplements during the first 8 weeks of consumption, a slight preference for the vanilla biscuit was manifested during the last 2 weeks of the study when women were given a choice of supplements. Women's potential reluctance to give negative ratings to supplement characteristics or its use (Adu‐Afarwuah et al., 2011) might have played a role in the reported acceptability during the first 8 weeks of the study. We therefore suggest to introduce a period in which a choice is offered to measure medium‐term acceptability. Offering a choice between supplements in an intervention trial or programme could also be an option, but more research is warranted to evaluate the added value, effect and practical feasibility.

The key limitation of this study is the potential for social desirability bias (Fisher, 1993). A mixed methods approach was adopted to mitigate potential bias of each single method and combined different approaches to triangulate findings and generate a more complete picture. Further, facilitators encouraged participants to speak openly and honestly in their local language, and they ensured the concept of confidentiality was well understood. Finally, we performed two home observations per household. The aim of this was to reduce change in behaviour that may have emerged during the first observation because an outsider was in the home. Although it is nonetheless possible that women may have altered their behaviour due to the presence of an observer (Langlois et al., 2020), Harvey, Olórtegui, Leontsini, and Winch (2009) argue that the impact on validity is often minimal and propose to validate conclusions by collecting data in multiple ways. It is worth discussing that we did not find a noticeable difference between the compliance measured by participants' answers and the home observations. This is in contrast to another study in Burkina Faso (Abbeddou et al., 2015) in which compliance was higher in methods that relied on self‐reporting. Although it is difficult to compare both studies, a possible explanation for the internal consistency between the two methods in this formative study could be that the MISAME study team is well known in the study area due to previous work. Their trustworthy reputation and the daily presence in the households could thus have lowered rates of misreporting.

For the planned efficacy study, the high acceptability and compliance rates reported in this preliminary research is a valuable result. Few women spoke however about explicit factors, such as palatability, flavour, form or packaging, that described why they liked or preferred a supplement. Despite the fact that our data collectors tried to trigger responses capturing ‘why’, the answers often remained minimal in terms of ‘I like it’, ‘I don't like the other supplement’, ‘you asked me to choose, otherwise both are the same’. Finally, the high compliance rates in this 10‐week home feeding trial are a good indicator for medium‐term acceptably, but it is worth mentioning that this is a relatively short period to assess behavioural fatigue that could arise when the supplement is introduced during pregnancy and lactation, with daily consumption for a period of 6 months or more. A specific study should be designed to assess behaviour over longer periods of time to draw conclusions on the long‐term acceptability and use of BEP supplements.

## 5 CONCLUSION

We conclude that both the lipid‐based peanut paste and vanilla biscuit were acceptable and likely to be well used in the future. Women were positive about the taste and portion size of the supplements and willing to continue supplementation. Emphasis on the health benefits, informing and engaging family members and carefully monitor potential sharing remain important factors to support acceptance and compliance. As social context and individual preferences vary by setting and population (Dewey, 2009; Manary, 2015), this study provides an example of how a mixed‐method assessment can provide useful information on the acceptability of nutritious food supplements in research and programming.

## CONFLICTS OF INTEREST

The authors declare that they have no conflicts of interest.

## CONTRIBUTIONS

The authors' responsibilities were as follows: BdK wrote the manuscript; SI, PK, SdP, JB, KM, LJ, BdK and KV designed the study and the protocol; LCT, BdK, KV, KM and LJ trained the field data collectors; MO, RG, BdK and KV directed the field data collection; SI, BdK, KV, KM and LJ analysed and interpreted the data; SI, PK and CL coordinated the implementation of the study and helped analysing and interpreting the data. All authors contributed substantially to the manuscript and approved the final version.

## Supporting information


**Table S1.** In‐depth interview guide for pregnant women
**Table S2**. Semi‐structured thematic FGD guide for pregnant women.

**Table S3.** Home activities during 12h observation (total number of observations)
