# Supplementary material for: Home consumption of two fortified balanced energy protein supplements by pregnant women in Burkina Faso
Source: Matern Child Nutr. 2021 Jan 6;17(3):e13134. doi: 10.1111/mcn.13134 (PMC8189188; doi:10.1111/mcn.13134)
Supplement: Supplementary file 1 — Table S1. In‐depth interview guide for pregnant women Table S2. Semi‐structured thematic FGD guide for pregnant women. [file MCN-17-e13134-s001.docx]

**Supplementary table 1**. In-depth interview guide for pregnant women.

| **Initial interview (week 1)** |
| --- |
| Role of pregnant women in the household:   - Daily activities and adaptations due to pregnancy - Announcement of pregnancy |
| Household food practices and beliefs:   - Food practices such as decision making, serving and sharing - Beliefs and traditions regarding foods in general and during pregnancy |
| Diet during pregnancy and lactation:   - Importance of diet during pregnancy and lactation - Dietary intake and behavior changes during pregnancy and lactation |
| Availability and access to supplements:   - Knowledge of supplements for pregnant women - Current intake and availability of vitamin supplements |
| Antenatal consultations (ANC):   - Attendance - Attitudes towards ANC |
| **Supplement-specific interview (week 4 and 8)** |
| Evaluation of the supplement:   - Appreciation in terms of taste, texture, smell and color - General appreciation: overall judgement, portion size, resemblance to other foods |
| User experience:   - Timing, food replacement, sharing and changes over time |
| Future use:   - Willingness to continue using the supplement for the duration of the pregnancy - Willingness to pay for the supplement |
| **Final interview (week 10)** |
| Product preference:   - Preferred choice for Peanut paste or Vanilla biscuit |
| User experience:   - Experience after using the supplements for a longer period in terms of general feeling, satiety, weight changes, etc. - Experience and perception of household and community members |
| Future use:   - Willingness and reasoning to continue supplementation during pregnancy and lactation - Opinion regarding having a choice between the supplements - Opinion regarding sharing in the future; and suggestions to prevent sharing |
| Distribution and information:   - Preferred distribution channel - Preferred information on the supplements - Opinion regarding the involvement of household and community members |
| Conclusion |

**Supplementary table 2**. Semi-structured thematic FGD guide for pregnant women.

| User experience of the two BEP supplements:   - Overall evaluation and perception over time - Use of the supplements and (changes in) meal pattern - Portion size - Timing of consumption - Opinion of other people |
| --- |
| Preference:   - Preference for one supplement over the other, incl. reasons - Opinion regarding having a choice between the two supplements   Future use:   - Intention and reasons for continued use in the future |
| Distribution and information:   - Preferred supply channel - Preferred information - Facilitating factors or barriers to use the supplements |
| Conclusion |
