# Supplementary material for: Home consumption of two fortified balanced energy protein supplements by pregnant women in Burkina Faso
Source: Matern Child Nutr. 2021 Jan 6;17(3):e13134. doi: 10.1111/mcn.13134 (PMC8189188; doi:10.1111/mcn.13134)
Supplement: Supplementary file 2 — Table S3. Home activities during 12h observation (total number of observations) [file MCN-17-e13134-s002.docx]

| Table 3. Home activities during 12h observation (total number of observations)† | | | | | | | |
| --- | --- | --- | --- | --- | --- | --- | --- |
|  | |  |  |  |  |  |  |
|  | |  |  |  |  |  |  |
| Other activities, n (%) | |  |  |  |  |  |  |
| Sleeping | 328 (5.7) | | 167 (5.8) | 161 (5.6) | 373 (6.5) | 186 (6.5) | 187 (6.5) |
| Lying | 179 (3.1) | | 66 (2.3) | 113 (3.9) | 191 (3.3) | 86 (3.0) | 105 (3.6) |
| Sitting quietly | 1177 (20.4) | | 562 (19.5) | 615 (21.4) | 1190 (20.7) | 649 (22.5) | 541 (18.8) |
| Standing | 325 (5.6) | | 149 (5.2) | 176 (6.1) | 313 (5.4) | 132 (4.6) | 181 (6.3) |
| Dressing | 32 (0.6) | | 17 (0.6) | 15 (0.5) | 29 (0.5) | 13 (0.5) | 16 (0.6) |
| Bathing/washing hands. face. hair | 94 (1.6) | | 43 (1.5) | 51 (1.8) | 101 (1.8) | 49 (1.7) | 52 (1.8) |
| Using toilet | 41 (0.7) | | 17 (0.6) | 24 (0.8) | 59 (1.0) | 28 (1.0) | 31 (1.1) |
| Reading | 0 (0.0) | | 0 (0.0) | 0 (0.0) | 24 (0.4) | 0 (0.0) | 24 (0.8) |
| Writing | 0 (0.0) | | 0 (0.0) | 0 (0.0) | 0 (0.0) | 0 (0.0) | 0 (0.0) |
| Praying | 12 (0.2) | | 8 (0.3) | 4 (0.1) | 28 (0.5) | 2 (0.1) | 26 (0.9) |
| Leaving the house / Being outside the house | 860 (14.9) | | 437 (15.2) | 423 (14.7) | 764 (13.3) | 283 (9.8) | 481 (16.7) |
| Housework (unspecified) | 499 (8.7) | | 231 (8.0) | 268 (9.3) | 478 (8.3) | 246 (8.5) | 232 (8.1) |
| Beating mats / carpets | 0 (0.0) | | 0 (0.0) | 0 (0.0) | 0 (0.0) | 0 (0.0) | 0 (0.0) |
| Mopping / washing floor | 3 (0.1) | | 3 (0.1) | 0 (0.0) | 8 (0.1) | 7 (0.2) | 1 (0.0) |
| Sweeping | 117 (2.0) | | 64 (2.2) | 53 (1.8) | 64 (1.1) | 25 (0.9) | 39 (1.4) |
| Washing the clothes | 171 (3.0) | | 131 (4.5) | 40 (1.4) | 155 (2.7) | 39 (1.4) | 116 (4.0) |
| Sewing/knitting | 64 (1.1) | | 64 (2.2) | 0 (0.0) | 0 (0.0) | 0 (0.0) | 0 (0.0) |
| Cleaning yard/garden | 6 (0.1) | | 6 (0.2) | 0 (0.0) | 26 (0.5) | 19 (0.7) | 7 (0.2) |
| Tending to animals (e.g. feeding, watering, grooming etc.) | 16 (0.3) | | 5 (0.2) | 11 (0.4) | 12 (0.2) | 6 (0.2) | 6 (0.2) |
| Caring for children (bathing, dressing, feeding, playing) | 159 (2.8) | | 47 (1.6) | 112 (3.9) | 199 (3.5) | 163 (5.7) | 36 (1.2) |
| Washing dishes | 218 (3.8) | | 89 (3.1) | 129 (4.5) | 147 (2.6) | 62 (2.2) | 85 (3.0) |
| Collecting water | 236 (4.1) | | 149 (5.2) | 87 (3.0) | 320 (5.6) | 207 (7.2) | 113 (3.9) |
| Collecting wood | 10 (0.2) | | 7 (0.2) | 3 (0.1) | 6 (0.1) | 5 (0.2) | 1 (0.0) |
| Chopping wood | 5 (0.1) | | 4 (0.1) | 1 (0.0) | 2 (0.0) | 0 (0.0) | 2 (0.1) |
| Light wood | 74 (1.3) | | 41 (1.4) | 33 (1.1) | 58 (1.0) | 26 (0.9) | 32 (1.1) |
| Pound | 68 (1.2) | | 20 (0.7) | 48 (1.7) | 39 (0.7) | 24 (0.8) | 15 (0.5) |
| Others (not specified in the list), n (%) | 163 (2.8) | | 73 (2.5) | 90 (3.1) | 248 (4.3) | 146 (5.1) | 102 (3.5) |

†each observation represents the registration of an activity at a 5-minute interval; some activities may thus have been recorded as more than one observation.
